# Supplementary material for: Detecting shortcut learning for fair medical AI using shortcut testing
Source: Nat Commun. 2023 Jul 18;14:4314. doi: 10.1038/s41467-023-39902-7 (PMC10354021; doi:10.1038/s41467-023-39902-7)
Supplement: Supplementary file 1 — Supplementary Information File [file 41467_2023_39902_MOESM1_ESM.pdf]

Supplement: Preventing Shortcut Learning for Fair Medical AI using Shortcut Testing

Alexander Brown, Nenad Tomasev, Jan Freyberg, Yuan Liu, Alan Karthikesalingam, Jessica Schrouff*

* corresponding author

Supplementary Figures

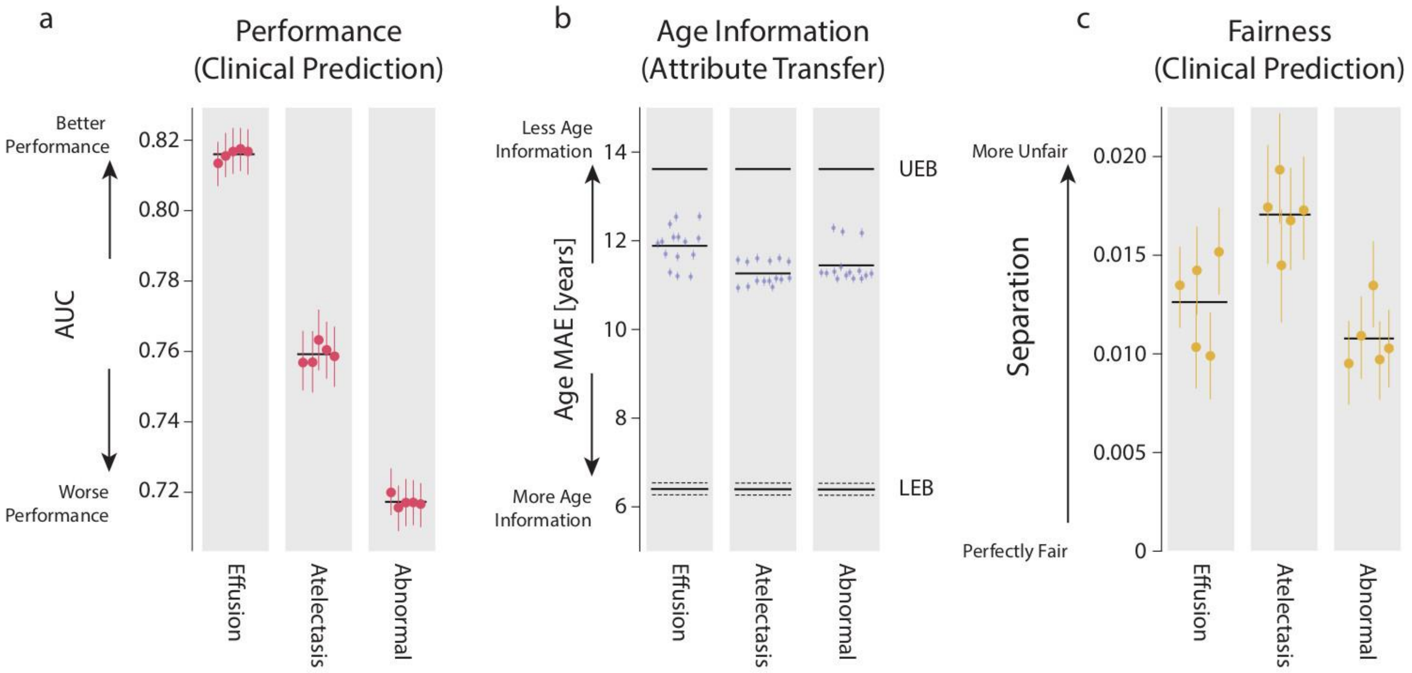

Supplementary Figure 1: Binary CXR prediction models. (a) Performance in terms of AUC for each task (Effusion, Atelectasis and Abnormal). (b) Age information encoded in each model in terms of age MAE. UEB: Upper Error Bound, LEB: Lower Error Bound, as determined experimentally. These bounds represent the limits for model age error in this dataset. The LEB is displayed as the mean and standard deviation across 5 technical replicates. (c) Fairness of each model in terms of separation, with 0 meaning a perfectly fair model (a separation value of 0.01 will correspond to a 10.5% change in model performance per decade; 0.02 will correspond to a 22.1% change per decade, see Methods).In all cases, each dot represents a different replicate of the model and error bars represent the population variability (95% bootstrapped confidence intervals, n=17,723 independent samples), with the average metric represented by a horizontal line. Source data are provided as a Source Data file.

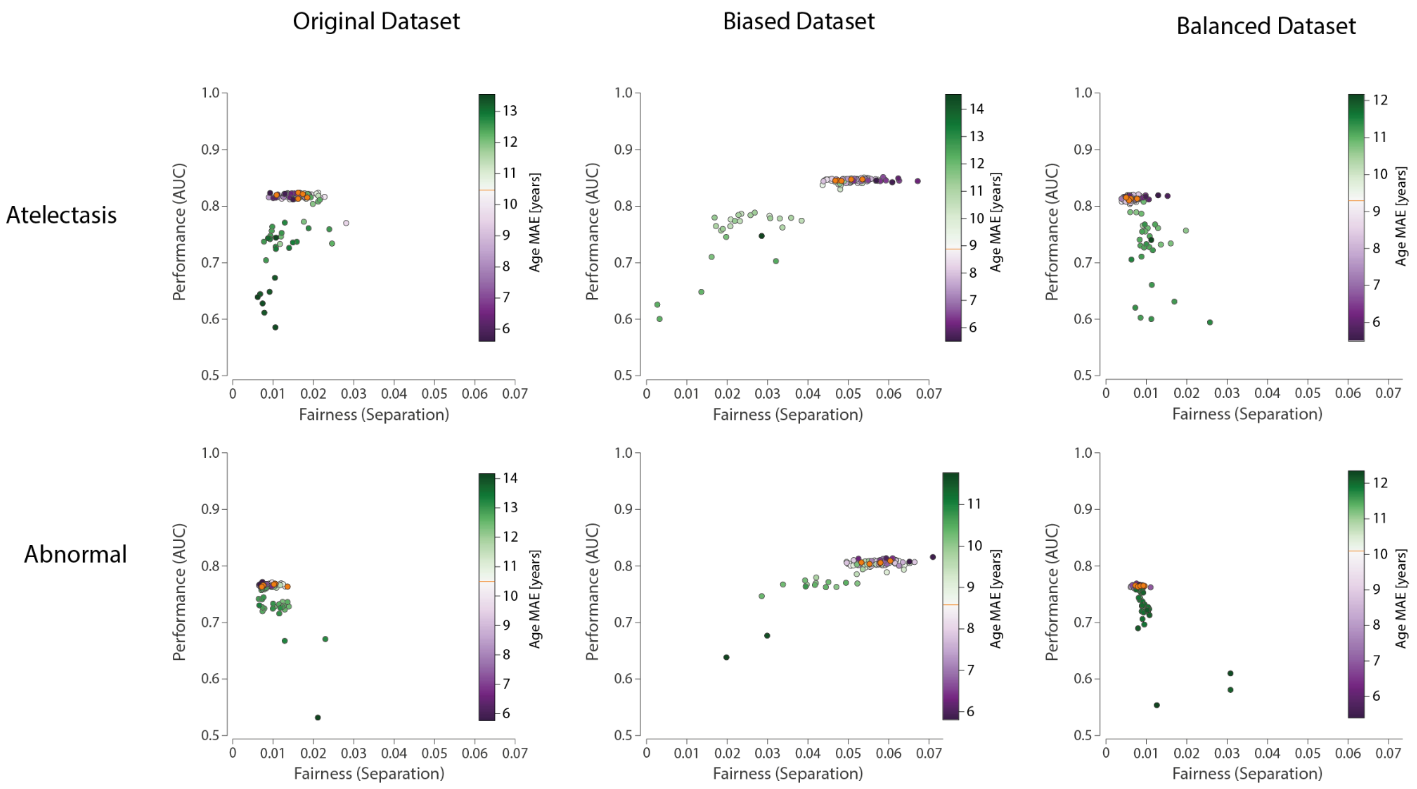

Supplementary Figure 2: Fairness-Performance results for other CXR labels. Separation is plotted against AUC, with the age performance of each model represented by the color as in Figure 3. Similar patterns may be observed, whereby inducing a bias in the training dataset results in much more unfair model performance, which can be ameliorated by gradient reversal (center column, green dots), or exacerbated by increasing the age representation (purple dots). In contrast, balancing the training dataset results in baseline models (orange) which are considerably fairer, and gradient reversal results in degraded model performance without further fairness improvement. Source data are provided as a Source Data file.

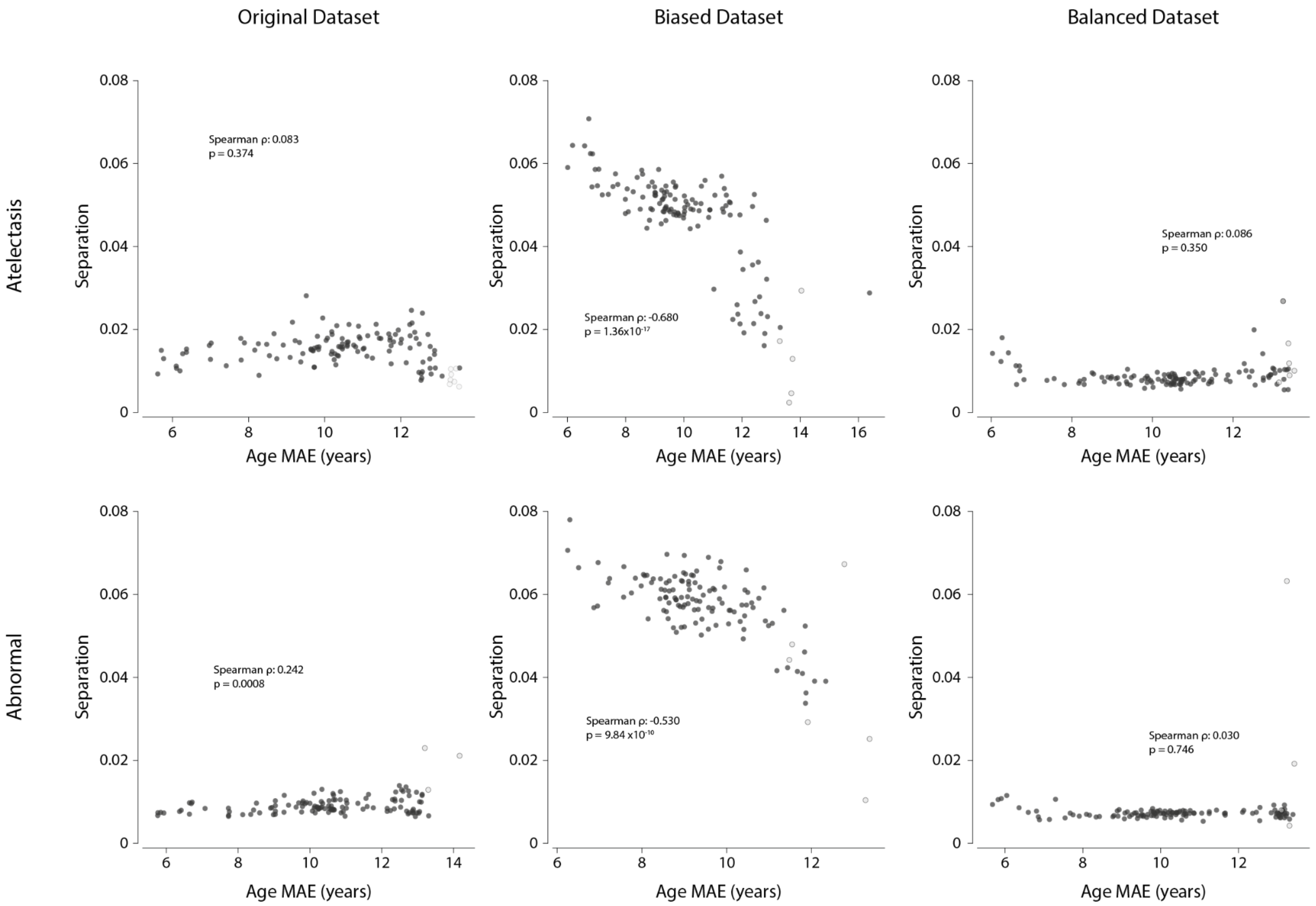

Supplementary Figure 3: ShorT analysis of original NIH and subsampled datasets for Atelectasis and Abnormal (the complement of the No Finding label). Biased datasets (middle column) result in significant dependence of fairness on age representation. In contrast, balanced datasets (right column), there is no such dependence. In the original dataset, there is no dependence of fairness on age representation for Atelectasis, however there is a significant positive correlation between fairness and age representation for the No Finding label. This implies that models which represent age more accurately (left) tend to be fairer (closer to 0 on the y axis). This may be explained by an underuse of age information for this particular dataset and task. For all plots, an AUC threshold was set at 0.7, with replicates with an AUC value less than this being excluded from the correlation analysis. We chose 0.7 as the threshold as the performance of baseline models was lower for Atelectasis and Abnormal labels (Supplementary Figure 1). One such replicate is not displayed on the Abnormal, Original Dataset plot, as it had a separation of > 0.1, and lies beyond the limits of the y axis. All tests are two-sided Spearman correlations. Source data are provided as a Source Data file.

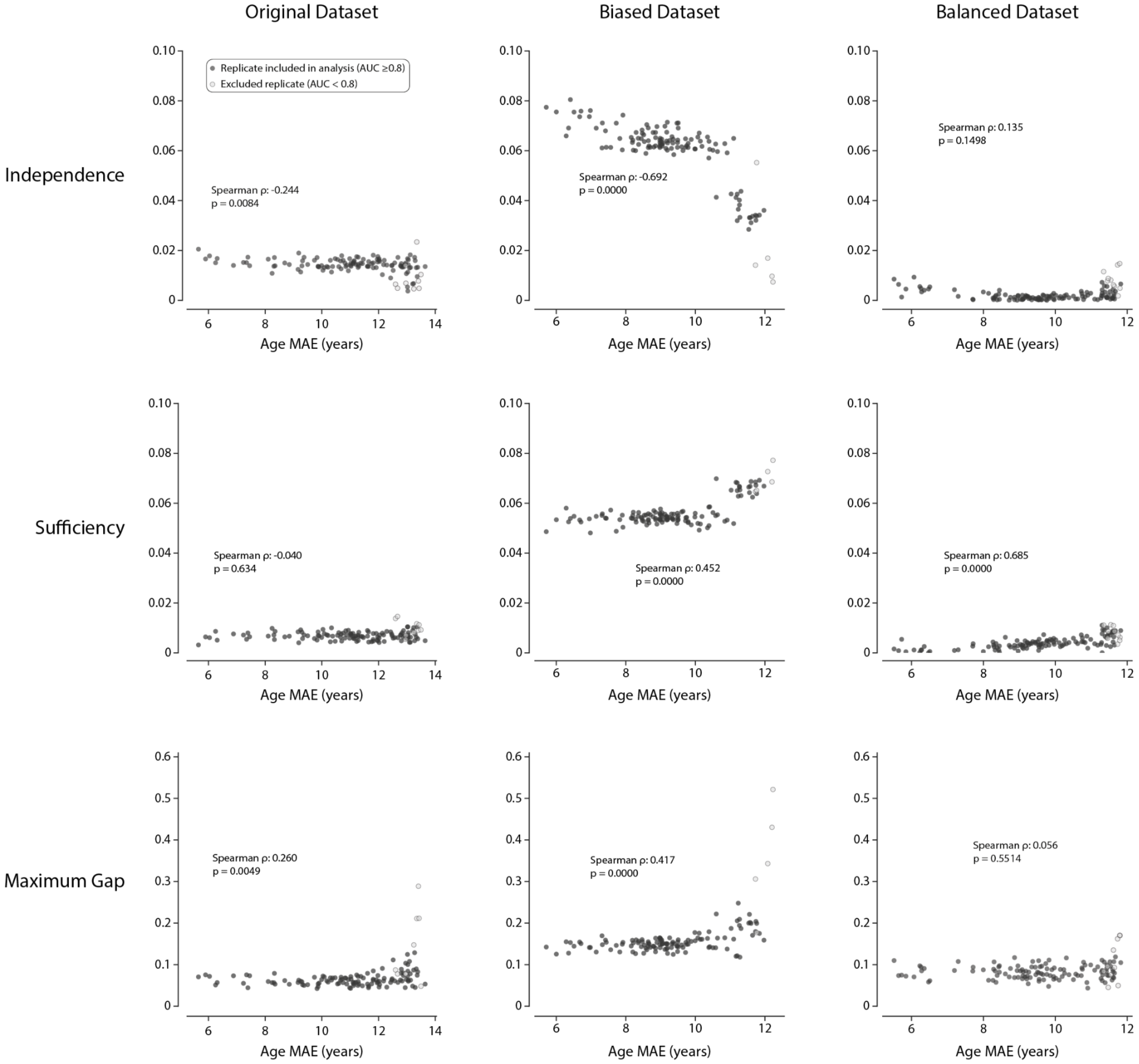

Supplementary Figure 4: ShorT analysis of original NIH and subsampled datasets for Effusion using different fairness metrics. (Top row) Independence, as computed by the coefficient of the logistic regression between the model's predictions and age. (Middle row) Sufficiency, as computed by the positive predictive value. (Bottom row) Maximum gap in performance across age subgroups, with age bucketed in [18,30), [30, 45), [45, 65) and [65, 100). For all plots, an AUC threshold was set at 0.8, with replicates with an AUC value less than this being excluded from the correlation analysis. All tests are two-sided Spearman correlations. Source data are provided as a Source Data file.

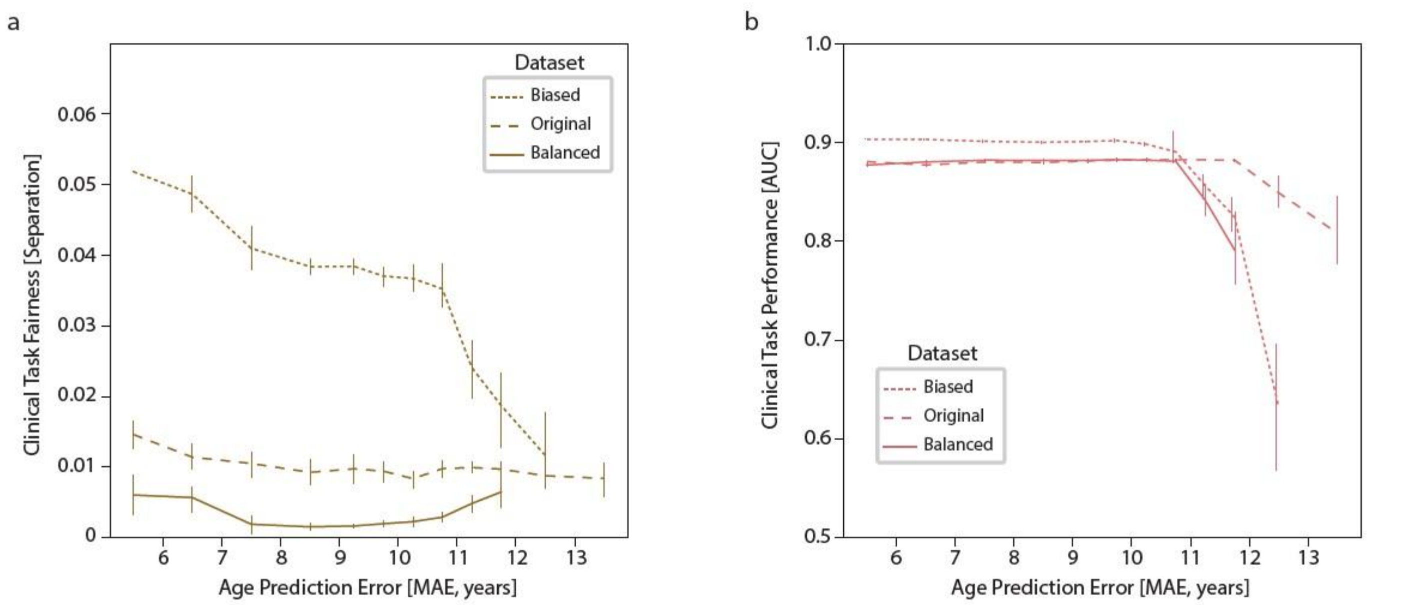

Supplementary Figure 5: Cross-Dataset Age comparison of fairness and performance at differing levels of age encoding in the Effusion task. (a) Fairness. Results from Figure 5 are displayed on a single graph, with replicates pooled according to predefined degrees of age encoding. In each degree of age encoding, we define its average (line) and estimate variability across models with bootstrapping (95% confidence intervals, error bars). For a given level of age encoding, models trained on the Balanced (solid line), Original (dashed line), or Biased (dotted line) datasets display vastly different fairness characteristics. (b) Age encoding vs Performance. At the same level of age encoding, performance is very similar for the Balanced and Original datasets, although the performance of the Balanced dataset drops off at higher age prediction errors. The Biased dataset results in a spuriously higher AUC due to cleaner class separation (see Supplementary Figure 6). Source data are provided as a Source Data file (see Figures 2c, 3c and 3f).

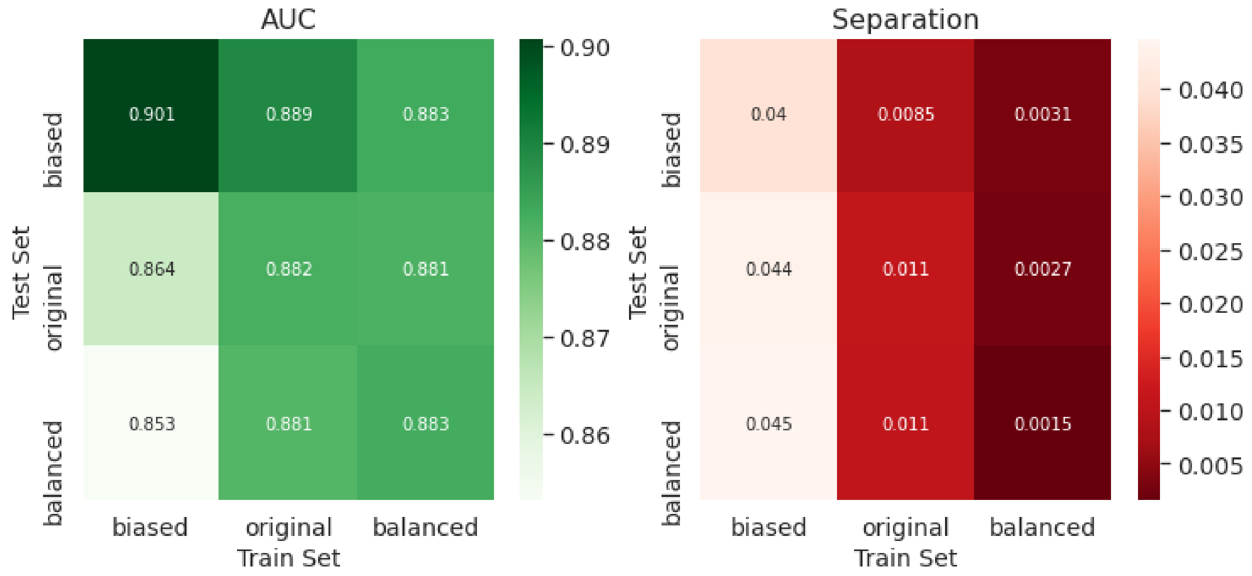

Supplementary Figure 6: Cross-dataset performance and fairness for the effusion prediction task. AUC and Separation are shown for baseline models (without an age prediction head) trained on biased, original, and balanced datasets (x axis), tested on all

three datasets. In-distribution results are located on the top-left to bottom-right diagonal. Note that the best performance is obtained in models trained on biased datasets, tested in-distribution; however, performance is degraded for out of distribution test sets, due to shortcut learning; this increase in performance is therefore spurious. Models trained on balanced datasets obtain similar performance results to those trained on the original dataset. However, separation is considerably improved in models trained on balanced data.

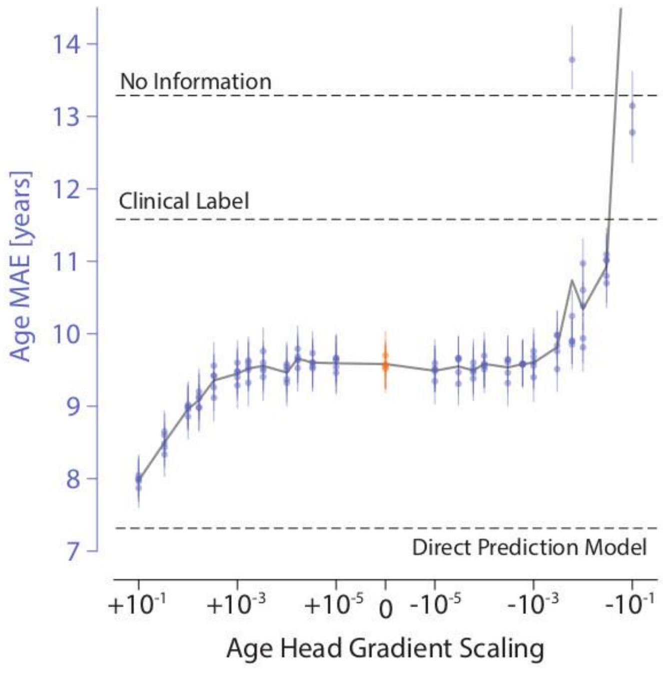

Supplementary Figure 7: Effect of age head gradient scaling on age representation for the dermatology example in figure 6. ShorT models covered a range of age prediction errors, although there appeared to be a wider plateau in the middle of the range of age head scaling values, over which age prediction error was quite similar to baseline. This plateau, as well as the wide range between the “Clinical Label” and “No Information” upper error bounds, likely occurs due to the richer soft labels used in this example, as well as due to the stronger dependence between age and condition probability for many (but not all) dermatological problems. Each dot represents a model trained (25 values of gradient scaling times 5 replicates), with error bars denoting 95% confidence intervals from bootstrapping examples (n= 1,925 independent patients) within a model. Source data are provided as a Source Data file.

Supplementary Methods

CXR Datasets

We use two CXR datasets, NIH CXR, and CheXpert. The NIH CXR Dataset is provided by the NIH Clinical Center and is available at https://nihcc.app.box.com/v/ChestXray-NIHCC. For experiments, images were first downsized to 448x448 pixels. We select the “Effusion”, “Atelectasis” and “No findings” (which we report as “Abnormal” for semantic consistency) labels provided with data as our binary outcomes, focussing on Effusion.

CheXpert is available at https://stanfordmlgroup.github.io/competitions/chexpert/. Demographic labels are available at https://stanfordaimi.azurewebsites.net/datasets/192ada7c-4d43-466e-b8bb-b81992bb80cf. Following[22], we focus on a binary distinction between Black and White patients, rather than treating race as a multi-class prediction task. Images were downsized to 448x448 pixels, and we select cardiomegaly as a binary outcome for reporting.

The demographic information for the NIH dataset is as follows, broken down by findings:

			Train	Tune
No Effusion	Female	Avg age	47.095458	44.460953
		N	26200	16698
	Male	Avg age	48.625099	44.311849
		N	32974	22931
Effusion	Female	Avg age	50.663645	48.79426
		N	3199	2683
	Male	Avg age	51.563483	48.064258
		N	3560	3875
No Atelectasis	Female	Avg age	47.10309	44.627401
		N	26792	17335
	Male	Avg age	48.547831	44.307013
		N	32751	23683
Atelectasis	Female	Avg age	51.395474	48.733138
		N	2607	2046
	Male	Avg age	52.059212	49.004483
		N	3783	3123
No Finding	Female	Avg age	46.252016	42.902625
		N	16991	9448
	Male	Avg age	47.98801	43.51865
		N	21268	12654
Finding	Female	Avg age	49.170374	47.113662
		N	12408	9933
	Male	Avg age	50.197891	46.048544
		N	15266	14152

Model Architectures

For the medical imaging models, we employ convolutional neural networks as image embedding models, followed by multi-layer perceptrons (MLPs) as classification models for both clinical classification and

age/race prediction. We use modified ResNet 101x3 architectures[1] pre-trained on the public Imagenet 21k dataset. Model architectures for image embedding and checkpoints are available on [tensorflow hub].

In the dermatology task, each clinical case includes 1 to 6 images. We average the embeddings across a clinical case before passing them to the MLP. All clinical classification MLPs have 2 layers, with 512 hidden units and ReLU activation, while all sensitive attribute MLPs have 3 layers, with 512 and 256 hidden units and ReLU activation.

In array pseudocode, the architecture follows:

```
# images: array of size (num_instances, height, width, 3)
# num_classes: 27 if dermatology, else 2
image_embeddings = resnet_101x3(images)
if dermatology:
 image_embeddings = image_embeddings.mean(axis='instance')
clinical_prediction = mlp(
   image_embeddings,
   layers=2,
   units=(512, num_classes),
   activations=('relu', 'softmax')
)
attribute_prediction = mlp(
   reverse_gradient(image_embeddings),
   layers=3,
   units=(512, 256, 1),
   activations=('relu', 'relu', None)
)
if age:
 loss = (cross_entropy(clinical_prediction, labels) + lambda *
        mse(attribute_prediction, sensitive_labels))
elif race:
 loss = (cross_entropy(clinical_prediction, labels) +
        lambda * binary_ce(attribute_prediction, sensitive_labels, from_logits=True))
```

Where the reverse_gradient is an operation that allows for gradient scaling and lambda is a hyper-parameter (positive or negative) that controls for the strength of the scaling. See the code available at https://github.com/google-research/google-research/tree/master/shortcut_testing for an example implementation of these different operations.

Hyperparameter Tuning and model selection

All models were tuned for batch size, learning rate, weight decay, and dropout in the penultimate layer before training. The same parameters were applied to models trained on each label in the CXR task.

	Batch Size	Learning Rate	Weight Decay	Dropout
Age Prediction	16	1×10^{-5}	1×10^{-7}	0
CXR Prediction	16	4×10^{-5}	1×10^{-6}	0.1
Age Transfer	8	3×10^{-3}	n/a	0

Models were trained for 17,500 epochs and the model with highest performance on the validation data was selected. The decision threshold for each model was based on the maximum F1-score observed on validation data.

Multitask Prediction

To adapt a single task prediction model to multitask prediction, we added a demographic (age prediction) head at the final layer of the base model. There are no hidden layers between the feature extractor and the condition output layer. However, the demographic head itself uses two fully connected hidden layers between the gradient reversal layer and the final age output layer, to provide the network with capacity during adversarial training.

Next, in order to approximately balance the losses between the age (mean square error) and condition (cross-entropy) heads, we down-weighted the regression loss by a factor of 100. We then tested further adjustments to this loss weighting using a grid search (in conjunction with a coarse gradient scaling parameter sweep). In our case, we found that simple balancing of losses was sufficient.

Once the loss weighting was established, this was fixed for all further experiments. We then swept over 25 values for scaling of the gradient updates from the demographic head, ranging from -0.1 to +0.1 (spaced exponentially). For each value of gradient scaling, 5 replicates were trained, resulting in 125 models per experiment.

For attribute transfer experiments, the feature extractor was frozen and then a linear demographic prediction head was applied and the model retrained to predict age. Hidden layers were not required in this simpler (single task) prediction setup; we found that the addition of one or two hidden layers made no material difference to our results.

Subsampling of training data

In order to produce datasets with a shift in the mean age between the ground truth classes, we use a logistic probability function, which defines the probability of an example being retained as a function of the age of the patient:

$$p_{retain} = m \div (1 + e^{-k(a-a_0)})$$

Where k is the slope of the function; a_0 is the midpoint of the probability function (the age at which the probability of being retained is 0.5); and m is a scale factor that increases the probability of retaining examples. This defines a probability of retaining a positive example; for negative examples (patients without the condition), we use $1\text{-}p_{retain}$

The following parameters were used to generate subsampled training sets. Since the process is stochastic, these were obtained by trial and error.

	k		a_0	m
	Biased	Balanced		
Effusion	0.14	-0.07	50	4
Atelectasis	0.12	-0.08	50	4
Abnormal	0.14	-0.065	50	4

The training sets generated using these parameters are described below. These perturbed datasets do not precisely match the desired shift in ages due to stochastic errors.

	Number of training examples			Positive Examples (% of training set)			Mean Age of Positive / Negative classes (years)			Performance (AUC) Fairness (Separation coefficient)		
	Original	Biased	Balanced	Original	Biased	Balanced	Original	Biased	Balanced	Original	Biased	Balanced
Effusion	65394	55634	61029	6731 (10.3%)	5612 (10.1%)	6421 (10.5%)	51.3 48.2	55.8 44.6	50.1 50.0	0.882 0.011	**0.901** 0.040	0.883 **0.002**
Atelectasis	65394	57800	59349	6354 (9.7%)	5592 (9.7%)	5959 (10.0%)	52.0 48.2	55.3 45.3	50.6 50.6	0.819 0.016	**0.846** 0.049	0.813 **0.006**
Abnormal	65394	55128	62294	27465 (42.0%)	22299 (40.5%)	22299 (40.5%)	50.0 47.5	55.1 44.2	49.2 49.0	0.766 0.010	**0.806** 0.057	0.765 **0.008**

Significance testing when comparing ShorT across datasets

Shortcut testing (ShorT) relies on calculating the correlation between the degree of age encoding and fairness metrics. To test that the ShorT statistics differ across datasets, we perform permutation tests of Spearman's rho across different versions of the training dataset. We calculate the true difference in correlation statistics, and compare it to an empirical null distribution of differences. The null distribution is simulated using bootstrapping. We combine the data points from the two groups, shuffle them, and randomly divide them into two groups. To calculate p-values, we compare the true difference to this null distribution.

For CXR, we find that differences are highly significant when comparing the original and biased datasets, and the original and balanced datasets (p = 1e-8; p = 1e-4, respectively), indicating that shortcutting happens significantly more with biased datasets, and significantly less with a balanced dataset.

Race in cardiomegaly models

To test ShorT in the context of a spurious attribute, we apply it to race in chest x-ray analysis. Following previous work[2], we analyze self-reported race in the CheXpert dataset as a binary task of predicting White and Black self-reported race from chest x-rays. We treat the Uncertain label for Cardiomegaly as negative.

The public validation set available for CheXpert only contains 9 individuals with self-reported Black race. Due to this small sample size, we instead randomly re-split the training data into new training (85%), validation (5%), and testing (10%).

This re-split has the following properties:

		Tune		Train	
		No Cardiomegaly	Cardiomegaly	No Cardiomegaly	Cardiomegaly
White	Female<br><br>Male	N = 4447<br>Avg Age = 64.9<br>N = 6652<br>Avg Age = 62.0	N = 491<br>Avg Age = 69.6<br>N = 979<br>Avg Age = 67.1	N = 40080<br>Avg Age = 64.9<br>N = 60011<br>Avg Age = 62.0	N = 4537<br>Avg Age = 70.3<br>N = 8427<br>Avg Age = 66.5
Black	Female<br><br>Male	N = 480<br>Avg Age = 58.6<br>N = 501<br>Avg Age = 57.8	N = 120<br>Avg Age = 61.6<br>N = 89<br>Avg Age = 58.2	N = 4140<br>Avg Age = 56.4<br>N = 4508<br>Avg Age = 54.5	N = 1104<br>Avg Age = 58.6<br>N = 1028<br>Avg Age = 53.0
Other Race	Female<br><br>Male	N = 3116<br>Avg Age = 57.7<br>N = 4454<br>Avg Age = 57.2	N = 420<br>Avg Age = 61.9<br>N = 609<br>Avg Age = 60.0	N = 28065<br>Avg Age = 57.5<br>N = 40125<br>Avg Age = 56.1	N = 3883<br>Avg Age = 63.2<br>N = 5381<br>Avg Age = 59.5

We focus on the cardiomegaly prediction, as the cardiomegaly label is imbalanced for race (prevalence for White patients was 11.5%, prevalence for Black patients was 19.8%). Similar to our age models, we train models to directly predict race to estimate the upper bound of performance on race (as per the AUROC on this binary prediction task). We then train models to predict both cardiomegaly and race, while sweeping over the gradient scale for the race prediction head. We set the weight of both heads as equal, as the scale of the loss is the same order of magnitude, and vary the gradient scale between -0.1 and +0.1 to match other experiments in the paper. Using an implementation inspired by Alabdulmohsin et al.[3], we estimate fairness via equalized odds.

Dermatology Dataset and experiments

For dermatology experiments, models are trained to predict 26 skin conditions with an additional "other" category to capture the long tail of conditions, as a multiclass prediction task, as described in [4]. Our approach differs slightly from previously published results, as we use a more modern architecture (ResNet 101x3 rather than Inception v4), and a slightly smaller training dataset. The commercial dataset used consists of teledermatology images with associated diagnoses obtained by labeling by multiple dermatologists. Unfortunately, this dataset is not available for public use.

We assess model performance for a single class by using binarised metrics. For AUC, we use the prediction score of the chosen class. For separation, we define positive predictions to be examples where the top ranking prediction score is for the chosen class. Using top-3 selection (i.e. a positive prediction is any example where the score for the chosen class is in the top-3 scores) did not change our results.

Dermatology Dataset - Demographics

Since the dermatological dataset is not publicly available, we report here the basic demographics of the training dataset used. This dataset comprises 12,027 cases obtained from teledermatology clinics in California and Hawaii:

Attribute		Percentage in training set
Race	American Indian / Alaska Native	0.83
	Asian	11.6
	Black / African American	5.99
	Hispanic / Latino	41.9
	Native Hawaiian / Pacific Islander	1.52
	White	35.5
	Not Specified	2.71
Gender	Male	38.1
	Female	61.9
Age	18-19	7.33
	20-29	22.4
	30-39	19.2
	40-49	16.8

	50-59	19.5
	60-69	11.6
	70-79	2.27
	80-89	0.782
	90+	0.191

Supplementary Note 1

Simulated data

We generated simulated data to assess the efficacy of ShorT. The data consisted of MNIST images[5] with the labels representing whether the number hand-written in the image was smaller than 5, or 5 and above. To these images, we added a small colored square at a random location. The color of the square (red or green) could be correlated with the label, and here plays the role of the sensitive attribute A. Noise was added to the image and the square as the tasks were straightforward. We hence obtain a data generating process that corresponds to Figure 1(b). As we control the data generating process, we are also able to generate counterfactual samples, i.e. images for which the color of the square has been switched.

We implemented ShorT with Tensorflow[6] v2 and Keras, using as feature extractor a small MLP of 3 dense layers with 10 units each. For gradient reversal, we added one more dense layer of size 2 before the attribute's output layer while the label was directly predicted from the feature extractor. Attribute encoding was assessed as the ROC AUC after training an output layer from a frozen feature extractor. Fairness was computed via equalized odds. Baseline model accuracy was between 0.8 and 0.86.

Further hyper-parameter selection was needed to balance the losses of the target (weight =1) and of the attribute (search between 0.5 and 1.0). The final value was selected as 0.75. We varied the correlation between Y and A such that a label of Y=0 was associated with a red square between 50 and 95% of the time (20 steps), while the label Y=1 was associated with a red square between 50 and 15% of the time (20 steps).

We observed that ShorT produces significant results for high correlations between Y and A (Supplementary Figure 8a). This corresponds to our observations with counterfactuals that, given the simplicity of the task, the model does not "need" to rely on the attribute for predictions if the correlation between A and Y is not high. Focussing on the low correlation setting, we uniformly sampled the correlations between A and Y in the 0.4-0.6 range (n=50) and assessed the number of significant results for ShorT (at a threshold of $p<0.05$, Bonferroni corrected). We note that only 3 instances lead to significant p-values for ShorT (i.e. 3/50=0.06 ≈ 0.05, Supplementary Figure 8b). Finally, we focussed on the high correlation setting and sampled uniformly in the 0.9-0.98 range for label Y=1 and in the 0.15-0.23 range for Y=0. Note that the asymmetry is needed to obtain unfairness based on equalized odds. In this case, we observe that ShorT correctly identifies

shortcutting in all instances (Supplementary Figure S8, 50/50), even after Bonferroni correction for multiple comparisons (50/50).

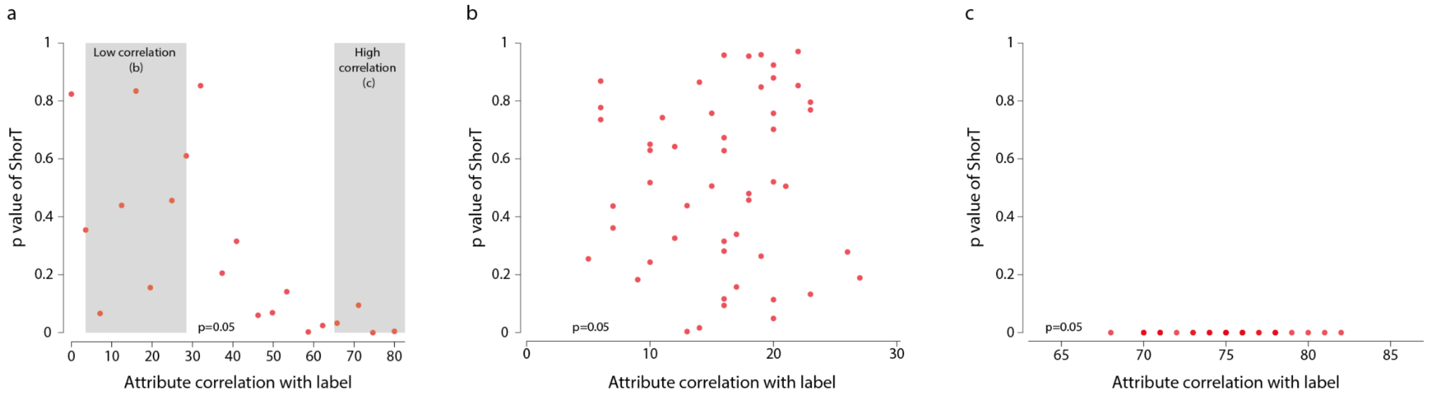

Figure S8: ShorT on simulated data. (a) Increasing the correlation between label and color in a consistent but asymmetric fashion leads to significant shortcutting for high values of the correlation. Each dot represents the p-value of ShorT computed based on a different combination of correlations. We focus on two areas (shaded on the plot): a low correlation setting (detailed in (b)) to assess type I error and a high correlation setting (detailed in (c)) to assess type II error. (b) For low values of the correlation and a small asymmetry (x-axis), we obtain a uniform distribution of ShorT p-values. (c) p-values are consistently lower than $p<0.05$ when the asymmetry is high and the correlation between A and Y is large. All tests are two-sided Spearman correlations, with p-values corrected for multiple comparisons using Bonferroni correction.

Supplementary Discussion

In our analysis, we have chosen to preserve age as a continuous variable, using logistic regression analysis to characterize the fairness properties of the model. This avoids the need for arbitrary quantization of the data. However, it does assume that discrepancies, where observed, will be monotonic - with weaker performance for either older or younger patients. In cases where we may expect bimodal or more complex distributions of fairness properties it might be more judicious to examine the model outputs rather than rely on particular formulations of fairness metrics. Distribution-free approaches [7–9], may be considered if no particular form of association can be expected, although these will in general be more limited in power and interpretability. Secondly, the use of a LR model requires a binarised outcome per example, and would be unsuitable for metrics such as prediction scores (continuous) or AUC (requires a set of observations). Alternative methods [10,11] may overcome some of these limitations, at the expense of interpretability. However, our framework does not require the use of a continuous attribute, and may be applied to binary or discrete variables, by substituting the model-based fairness metrics for conventional definitions.

Supplementary References

1. Kolesnikov, A. *et al.* Big transfer (BiT): General visual representation learning. in *Computer Vision – ECCV 2020* 491–507 (Springer International Publishing, 2020).
2. Gichoya, J. W. *et al.* AI recognition of patient race in medical imaging: a modelling study. *Lancet Digit Health* **4**, e406–e414 (2022).
3. Alabdulmohsin, I. M. & Lucic, M. A Near-Optimal Algorithm for Debiasing Trained Machine Learning Models. in *Advances in Neural Information Processing Systems* (eds. Ranzato, M., Beygelzimer, A., Dauphin, Y., Liang, P. S. & Vaughan, J. W.) vol. 34 8072–8084 (Curran Associates, Inc., 2021).
4. Liu, Y. *et al.* A deep learning system for differential diagnosis of skin diseases. *Nat. Med.* **26**, 900–908 (2020).
5. Deng, L. The MNIST Database of Handwritten Digit Images for Machine Learning Research [Best of the Web]. *IEEE Signal Process. Mag.* **29**, 141–142 (2012).
6. Martín Abadi *et al.* TensorFlow: Large-Scale Machine Learning on Heterogeneous Systems. Preprint at https://www.tensorflow.org/ (2015).
7. Blum, J. R., Kiefer, J. & Rosenblatt, M. Distribution Free Tests of Independence Based on the Sample Distribution Function. *Ann. Math. Stat.* **32**, 485–498 (1961).
8. Gretton, A., Bousquet, O., Smola, A. & Scholkopf, B. Measuring Statistical Dependence with Hilbert-Schmidt Norms. *Plan. Perspect.* **63**, 77 (2005).
9. Sricharan, K., Raich, R. & Hero, A. O., III. Empirical estimation of entropy functionals with confidence. *arXiv [math.ST]* (2010).
10. Miller, A. C., Gatys, L. A., Futoma, J. & Fox, E. Model-based metrics: Sample-efficient estimates of predictive model subpopulation performance. **149**, 308–336 (06--07 Aug 2021).
11. Estiri, H. *et al.* An objective framework for evaluating unrecognized bias in medical AI models predicting COVID-19 outcomes. *J. Am. Med. Inform. Assoc.* **29**, 1334–1341 (2022).
